# Recent Advances in Ionic Liquids and Ionic Liquid-Functionalized Graphene: Catalytic Application and Environmental Remediation

**DOI:** 10.3390/ijerph19137584

**Published:** 2022-06-21

**Authors:** Han Zhou, Shaoyuan Bai, Yanan Zhang, Dandan Xu, Mei Wang

**Affiliations:** 1 College of Environmental Science and Engineering and Guangxi Key Laboratory of Environmental Pollution Control Theory and Technology, Guilin University of Technology, Guilin 541004, China; zhouhanwy@163.com (H.Z.); baisy@glut.edu.cn (S.B.); zyanan@glut.edu.cn (Y.Z.); 2 Collaborative Innovation Center for Water Pollution Control and Water Safety in Karst Area, Guilin University of Technology, Guilin 541004, China; 3 Heng Sheng Water Environment Treatment Co., Ltd., Guilin 541100, China; 19167545350@163.com

**Keywords:** ionic liquids, graphene, IL-functionalized graphene, catalysis, environment

## Abstract

Applications of ionic liquids (ILs) for the modification physicochemical properties of porous materials have been extensively studied with respect to various applications based on the understanding and development of properties of ILs. In this review, IL–graphene composites are discussed and provided a perspective of composites of IL. IL has been used as a medium to improve the dispersibility of graphene, and the resulting composite material shows excellent performance in gas separation and catalysis during environmental treatment. The applications of ILs and IL–functionalized graphene are discussed in detail with the actual environmental issues, and the main challenges and opportunities for possible future applications are summarized.

## 1. Introduction

Increasing concentration and variety of environmental pollutants is driving the development of “green materials”. An IL-porous material composite has great potential for gas separation, catalysis, water treatment, and so on due to the combined advantages of both materials. Herein, we present a comprehensive overview of the recent advances in the applications of ILs and IL-functionalized graphene. Graphene is a novel two-dimensional carbon nanostructure, where a single layer of carbon atoms are arranged in a hexagonal lattice, resulting in a planar structure that leads to high specific surface area (monolayer graphene is 2630 m^2^/g). The unique physical and chemical properties of graphene make it an ideal adsorbent for adsorbates [1]. However, due to the tendency of graphene to agglomerate, the loss in each step of its production is significant, limiting its practical application. Fortunately, targeted covalent functionalization can enhance the performance of the material and expand its scope of application. Hence, the synthesis of functionalized graphene is an effective method and an important goal for practical application of the material. Among different guest molecules, ILs provide a high degree of flexibility due to the unlimited possibility of theoretical cation–anion combinations, high thermal/chemical stabilities, and low vapor pressures. The selection of required functional groups from the different cation–anion combinations of IL can help in adjusting the position of adsorption on the surface of graphene material. In terms of modifying the physicochemical properties of a pristine host material, ILs are one of the most promising materials. Rational designing of ILs provides multiple options for combining them with graphene. Understanding the effect of specific functionalization via rational selection of ILs can result in direct adjustment of the selectivity of materials towards a particular molecule. Presently, this aspect has not been paid attention in many reviews. In this paper, the removal of ILs is briefly summarized and explored. ILs have found wide application in environmental remediation due to their green nature. Some results have shown that ILs have sterilization and disinfection or herbicidal activity. They can be toxic when released into the environment [2]. Understanding the removal technology of ILs is also important.

## 2. Ionic Liquids

ILs are molten salts in the liquid phase at room temperature, are organic salts with a low melting point (<100 °C), and are commonly used as solvents. Most ILs have more environmentally friendly production pathways compared to conventional solvents and thus can be considered as alternative “green solvents” to volatile organic compounds (VOCs). Among different molecules, ILs provide a high degree of flexibility, high thermal/chemical stabilities, and low vapor pressures. Based on the type of cations involved, ILs can be mainly divided into the following four categories: imidazolium-based ILs, pyridinium-based ILs, quaternary ammonium-based ILs, and quaternary phosphonium-based ILs.

In spite of its late start, research on ILs in China has developed rapidly. It has unique advantages in the fields of gas–liquid separation, electrochemistry, organic reaction, etc. [3]. Extensive research has been carried out on the amalgamation of IL technology with various traditional technologies. Functionalized ILs having cations and anions which target specific organic moieties are designed and synthesized as per the requirements. Furthermore, quantum chemical calculations and molecular dynamics simulations have been utilized to control properties of ILs such as thermal stability, viscosity, surface tension, etc. with specific functional groups.

Other than the nature and size of anions and cations, the physical and chemical properties of ILs also depend on the balance between Coulomb and van der Waals interactions, as well as hydrogen bonding and π-π interactions. ILs are considered to be promising adsorbents for inorganic or organic pollutants due to their affinity for the target molecules. Low coordination ability, low interfacial tension and interfacial energy, and their higher degree of orderliness due to ease of formation of hydrogen bonds make them attractive both as media and templates during the preparation of nanomaterials with special morphologies. Therefore, the study of ILs is of great significance not only from the basic theoretical point of view but also regarding the aspect of practical applications.

### Characteristics of ILs

The typical characteristics and unique advantages of ILs in environmental remediation are shown in: in water environmental restoration, it has the characteristics of high separation efficiency, no secondary pollution and less consumables; In soil remediation, it can effectively weaken the toxicity of pollutants; In atmospheric remediation, the elimination of pollutants can sometimes be accompanied by their resourceful use. The future development trend of ILs in green preparation and environmental remediation.

## 3. Application of ILs

### 3.1. The Restoration of Water Environments

ILs have been applied as a green alternative for the treatment of environmental pollution. Low toxicity, environment-friendliness, high extraction efficiency, possibility of reuse, low energy consumption, and so on are some of the unique advantages offered by ILs over the traditional treatment methods for environmental remediation. The partition coefficient between ILs and water varies with the change of pH, offering the possibility of separation and enrichment of heavy metals as well as recovery from the IL medium and subsequent reuse. The removal efficiency of heavy metals such as cadmium, copper, and zinc by ILs reaches 90% to 99% [4]. In 2003, Wei et al. [5] successfully extracted Hg^2+^, Pb^2+^, and Cd^2+^, from the aqueous phase into 1-butyl-3-methylimidazolium hexafluorophosphate using dithizone as the chelating agent. Two imidazolium-based cationic polymeric nanotraps based on tetraphenylmethane building blocks (CPN-tpm) with different counterions were rationally designed and constructed for the efficient removal of Cr_2_O_7_^2^^−^ from wastewater. Such nanotraps not only offer a facile and efficient approach to enhance the adsorption performance of a cationic polymer towards Cr_2_O_7_^2^^−^ by simple exchange of the counter anion, but they also pave the way for the development of cationic polymeric nanotraps as a new type of adsorbent for wastewater treatment [6].

In the recent years, scientists have found that the performance and adsorption effect of graphene can be improved by loading ILs onto the surface of graphene. In 2013, Rajesh et al. [7] studied the removal of hexavalent chromium from wastewater by a composite formed by quaternary ammonium ILs and exfoliated graphene oxide (EGO). From the environmental point of view, regeneration of the adsorbents is possible via reduction of dichromate ions to relatively benign Cr^3+^. Tetraoctylammonium bromide (IL)-impregnated adsorbent showed higher adsorption capacity and faster adsorption kinetics in practical wastewater samples. In 2017, Nasrollahpour et al. [8] established a new method for the separation and enrichment of mercury by dispersion solid phase microextraction of magnetic graphene oxide nanoparticles modified by ILs. Due to high water solubility making it difficult to separate GO from water, the application of GO as an adsorbent from the aqueous phase is limited, but the introduction of magnetism into graphene oxide can effectively eliminate this limitation.

**Figure 1:**
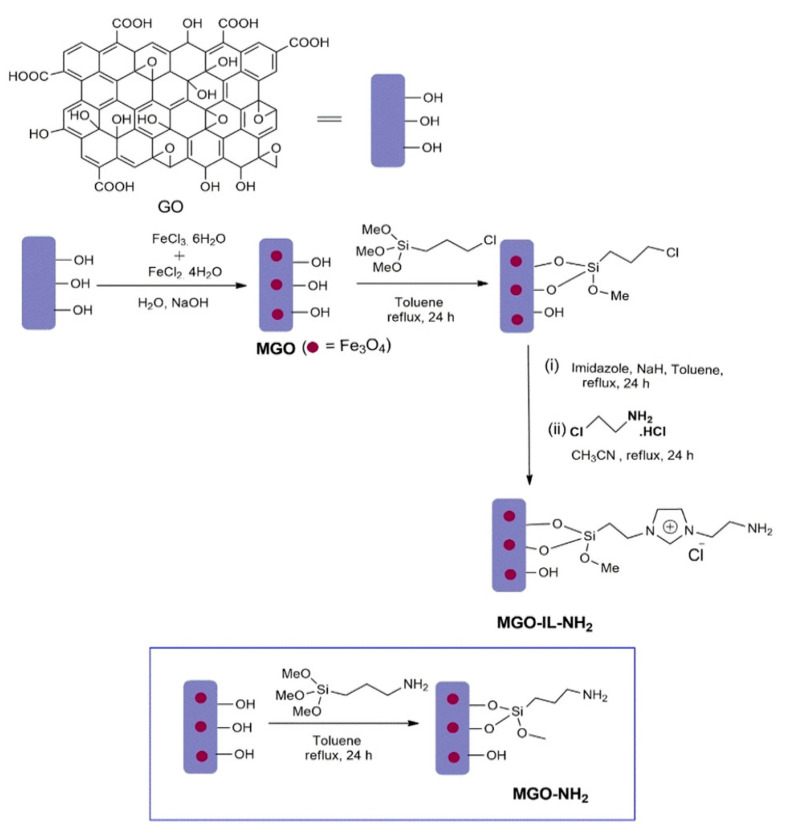
Schematic diagram for the preparation of magnetic graphene oxide-supported IL (MGO−IL−NH_2_).

Bazrafshan et al. [9] used nanocomposites consisting of 1-vinyl-3-butylimidazolium hexafluorophosphate-modified magnetic activated carbon (IL@mAC) to remove tetracycline from wastewater. The highest removal efficiency of ~88% was obtained under the conditions of pH 7, 303 K reaction temperature, 0.06 g/kg dosage, and 135 min reaction time. IL magnetic graphene has been used as an adsorbent to enrich fluoroquinolone antibiotics in water samples. The mechanism of IL remediation in water body is mainly manifested in the principle of extraction. It can also be used as an adsorbent for magnetic solid-phase extraction and combined with ultra-high-performance liquid chromatography tandem mass spectrometry to detect sulfonamides in real water samples. GO was synthesized by a modified Hummers’ method. Thereafter, magnetic nanoparticles (Fe_3_O_4)_ were added to the surface of the GO using the chemical deposition method. The resulting magnetic graphene oxide (MGO) was functionalized with imidazole by the reaction of MGO with 3-chloropropyltrimeth-oxysilane followed by N-alkylation of imidazolium salt. Finally, the quaternization of the resultant product was achieved by the reaction of the resultant with 2-chloroethylamine in order to obtain the final magnetic graphene oxide-supported amine-functionalized IL (MGO−IL−NH_2_) (Figure 1) [10].

### 3.2. Application in Catalytic Reactions

**Figure 2:**
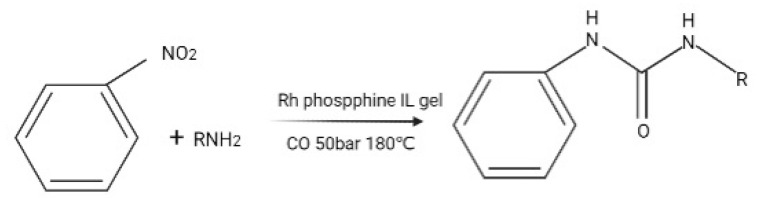
Carbonylation of amines and nitrobenzene catalyzed by metal phosphine-doped IL gels.

ILs as catalysts and alternative reaction media have become subjects of mainstream research in the field of synthetic organic chemistry. They have been used in various types of organic reactions, nanoparticles synthesis, and enzyme reactions [11,12]. ILs act as both a solvent and a catalyst in alkylation. Movahed et al. [13] modified graphene oxide by silylation and then supported it with ILs to synthesize the IL-functionalized graphene oxide-supported nitrogen heterocyclic carbene palladium complex NHC-Pd/GO-IL. The catalyst showed high catalytic activity for the Suzuki coupling reaction of aryl halide and arylboric acid in ethanol and aqueous solution, and the catalyst can be reused. Protic ILs have been used as acid catalysts to hydrolyze and dehydrate carbohydrates derived from biomass into renewable chemical platforms such as 5-hydroxymethylfurfural (HMF), levulinic acid (LA), and furfural (FUR) [14]. There are various means of loading ILs onto different carrier materials, such as magnetic nanoparticles [15,16], graphene [17], molecular sieves [17], metal skeletons, etc. Various types of supported functional ILs (SFILs) have been prepared, which, in addition to providing a good ionic environment, form strong interactions with the reaction substrate, resulting in better catalytic effects. Photocatalytic methods are a promising technology for pollutant treatment because the catalyst can use sunlight as an energy source to initiate and promote the degradation of organic pollutants. TiO_2_ is currently the most commonly used photocatalyst for decomposing water, which provides better activity in removing organic pollutants and shows more favorable mineralization level, thus preventing the accumulation of harmful organic pollutants in water. Due to the surface limitations and inadequate adsorption capacity of TiO_2_ resulting from its nonporous nature, for more effective removal of PCBs from wastewater treatment plants, it is useful to incorporate photocatalysts into porous supports in order to facilitate the efficient absorption of target environmental pollutants [18]. At present, TiO_2_ is widely used as a photocatalyst for water decomposition. Pt is loaded onto the surface of TiO_2_, and dyes and ILs are loaded with ethanol and acetone as solvents to improve the catalytic performance of Pt/TiO_2_ for hydrogen production [19]. Supported IL catalysis (SILC) was applied to a hydrogenation reaction [20], which reduced the amount of IL phase and made the fixed-bed technology possible. The obtained catalyst showed high activity and excellent stability. Based on the high charge of ionic phases, these ionic phases are ideal for biphasic reactions with organic substrates and allow for easy separation of the catalysts. For example, see Figure 2.

### 3.3. Application in Electrochemistry

**Figure 3:**
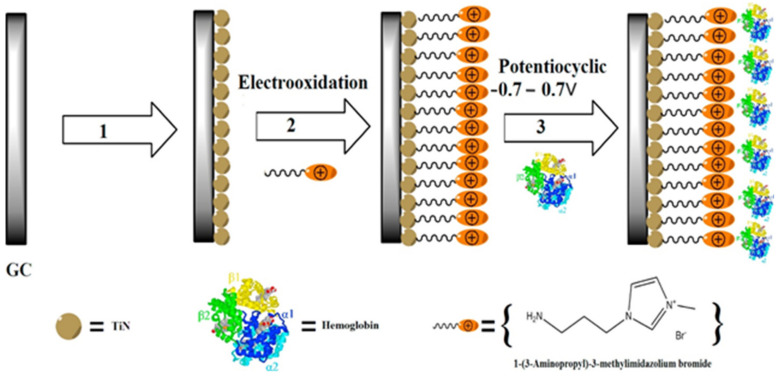
Schematic illustration of the fabrication process of GC/TiNnp/NH_2_−IL/Hb [24].

ILs can be used as electrolytes inside graphene electrodes due to its high ionic conductivity and wide electrochemical window [21], or they can be used in the preparation of carbon-based electrodes [22]. The addition of ILs could bring their unique advantages to the electrolyte membrane. They are proven to be efficient tools to improve the dispersion of materials when agglomeration is a problem. Nanomaterials have small volumes and large specific surface areas. Hybrids of ILs and nanoparticles can improve the performance of an electrolyte, resulting in a higher diffusion coefficient, higher conductivity, and better thermal and electrochemical stability. The combination of carbon nanomaterials and metallic nanomaterials [23,24] with ILs has become the research focus for the new generation of electrochemical sensors (Figure 3). Zhang et al. [25] reported the synthesis of core-shell Au@Pd nanoparticles smaller than 3 nm in the presence of 1-(2-aminoethyl)-3-methy-imidazolum tetrafluoroborate (Au/Pd molar ratio = 1). The IL-derived core-shell Au@Pd nanoparticles represent a method for the preparation of efficient Pd-based anodic electrocatalysts.

Electrochemical tests show that ILs improve the electrical conductivity of organic electrolytes, thus improving the cycle performance of electrochemical capacitors and reducing internal resistance [11]. The addition of ILs can improve the water absorption, the number of movable ions, and the electrical conductivity of a sensing film. Various π-bond-conjugated polymers such as polyaniline, polypyrrole, and polythiophene are widely used in batteries, capacitors, photovoltaic cells, and other electrochemical devices, which require electrolytes with high conductivity, wide electrochemical windows, high electron transfer rates, and low viscosity. However, general electrolytes do not meet the requirements, leading to shorter device lives. Therefore, ILs in combination with various π-bond-conjugated polymers are used to prolong the lives of electrochemical devices. ILs also play a strong catalytic role in the modification and electron transfer process, which is of great significance for electrochemistry.

## 4. IL-Functionalized Graphene

**Figure 4:**
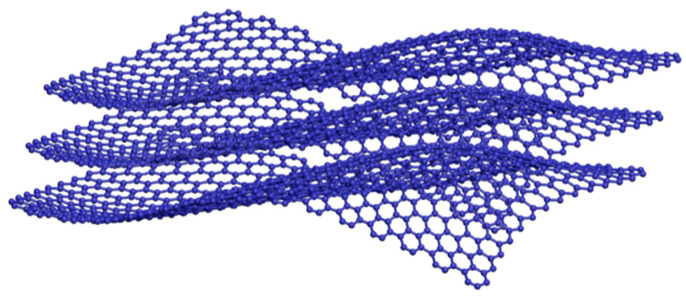
The graphene layers.

Graphene is a carbonaceous material with a two-dimensional honeycomb lattice structure in which sp^2^ hybrid monolayer carbon atoms are closely packed in a hexagonal manner. Its basic structural unit is a stable six-membered benzene ring. At present, the most widely used method to prepare graphene is to oxidize natural graphite. There are two main ways to obtain graphene. One is to reduce graphene oxide to graphene by using strong reducing agents (such as hydrazine hydrate, caffeic acid, and vitamin C). The other is thermal reduction of graphene oxide, which can be further subdivided into thermal-stripping [26] and microwave-stripping reduction. Thermally exfoliated graphene oxide (TEGO) is obtained by the thermal-stripping reduction of graphene oxide by removing various oxygen-containing groups (−OH, −COOH, C=O) in graphene oxide by heating. Microwave-exfoliated graphene oxide removes the oxygen-containing functional groups in graphene oxide by thermal shock produced by high-power microwaves in a short time. The electrochemical performance of graphene mainly depends on its lamellar arrangement and structure (Figure 4). Graphene nanoplatelets are easy to accumulate after chemical oxidation and reduction synthesis. The surface modification and structure adjustment of graphene is a hot spot in the research of graphene carbonaceous enrichment materials. The results show that graphene modified by ILs not only has stable structure but also has a high charge transfer rate, which is beneficial to the enhancement of electrochemical performance [27].

The modification of physical and chemical properties of porous materials using ILs has been extensively studied. It also has great potential in gas adsorption and separation, catalysis, water treatment, electrochemistry, etc. ILs are good solvents as well as modifiers [28]. Because the agglomeration of graphene nanosheets due to van der Waals interactions leads to lower specific surface area and weak electronic conductivity, graphene is modified by ILs, and the interaction between graphene and an IL is π-π and cation-π. Previous studies have shown that IL is used in the synthesis of functionalized graphene and plays a crucial role in controlling the spacing between graphene layers during the direct reduction of graphene oxide. The resulting hybrid materials, generally called graphene–IL layered films, contained IL molecules between graphene layers [29]. ILs can be better inserted between graphene layers and also adsorbed onto the surface, thus increasing the interlamellar spacing of graphene, which results in weakened van der Waals forces between the graphene layers and thereby effectively prevents the easy agglomeration of graphene. They can also improve the hydrophilicity of graphene to solve the shortcomings of uneven distribution. [30]. In a study by Xiao et al., ILs were used as stabilizers to obtain IL/graphene hybrid materials during graphite exfoliation [31]. Supported functional ILs have advantages of both ILs and carriers, and the synergistic effects of the two components greatly improve the conversion effect, which is more environmentally friendly and efficient and thereby has attracted increased attention.

### 4.1. ILs Enhance the Dispersion of Graphene

Xu et al. [32] successfully prepared imidazolium IL-functionalized GO catalysts. Imidazole-based IL was immobilized on the surface of GO material by covalent condensation between the hydroxyl group of GO and the alkoxyl group of functionalized IL.

The polydispersed lamellar graphene structure was obtained by nucleophilic ring opening of epoxy functional groups on the surface of GO by amine functional groups on the ILs [33,34]. Yang et al. [35] realized the covalent bond modification of GO by using a nucleophilic substitution reaction of amine-terminated 1-(3-aminopropyl)-3-methylimidazolium bromide (IL-NH_2_). GO and ILs containing amino groups can be converted into three-dimensional materials by a one-step hydrothermal method. Chen et al. [36] prepared graphene nanocomposites with good dispersion in water via hydrogen bonding between -NH_2_ and -OH groups in doxorubicin hydrochloride (DXR) and -COOH and -OH groups on graphene oxide.

The structures of GO and graphene contain large aromatic rings, which are easy to stack with modifiers containing aromatic rings or pyridine. Deka et al. [37] modified graphene prepared by vapor deposition by loading positively and negatively charged carbon nanoparticles onto graphene and obtained significantly improved electrical conductivity of graphene. The carboxyl groups on GO and graphene can become negatively charged under certain conditions and thus can interact with positive ion modifiers for the modification of graphene or the preparation of graphene/polymer nanocomposites. Choi et al. [38] obtained non-covalently modified graphene of polystyrene via electrostatic interaction between the amine group and the residual carboxyl group on the surface of graphene. Graphene can be easily dispersed in hydrophilic imidazolium ILs or pyridinium-based ILs by mechanical grinding to form a stable system when the concentration reaches 7.0 mg/mL in the absence of any surfactant treatment [39]. It has been reported that the low-temperature physical properties of ILs can be significantly improved through the interaction between ILs and graphene. There are many interactions between imidazole-based ILs and graphene, including the oxidation of oxygen groups on the surface of graphene, π-π conjugation effect between graphene layer and imidazole ring, and cation-π interaction. For example, imidazolium ILs were used as a coupling agent between graphene and polymethylmethacrylate, which promoted the dispersion of graphene and improved the electrical conductivity of the composites by solution formation and in situ polymerization. Wang et al. [40] functionalized multi-walled carbon nanotubes with ILs containing amine groups and then loaded Au nanoparticles onto the surface to prepare gold nanoparticles with small size and good dispersion. Compared to a glassy carbon electrode, the electrode consisting of the catalyst has better oxygen reduction performance. This is mainly due to the modification of carbon nanotubes by ILs.

### 4.2. ILs Improve the Conductivity of Graphene

The high conductivity and wide electrochemical window of ILs have been noticed for a long time. ILs and mixtures containing ILs are electrolytes. Under an applied electric field, the anions and cations of ILs will migrate to the surface of graphite rods as positive and negative electrodes. Because the electric field force will balance the force between the graphite layers, the peeling phenomenon of graphite rods will occur in the positive and negative electrodes. The size of graphene sheet varies from nanometer to micron, and it does not introduce a large number of defects on the surface of graphene, but sometimes are accompanied by redox reactions, which has an effect on exfoliation [41].

At present, the research on IL-GO composite systems is mainly concentrated in the fields of batteries, electrolytes, and capacitors [42,43,44]. The first application of an IL-modified electrode was reported by Marken et al., in the late 1990s.

Kim J. et al. obtained improved electrochemical performance of graphene nanosheets modified by 1-ethyl-3-methylimidazolium hexafluorophosphate. Ke-Jing Huang et al. designed a novel electrochemical sensor based on a graphene sheet (GR-NH_2_) and gold nanoparticles (AuNPs) composite-modified electrode based on amino-functionalized ILs. CILE was fabricated by using 1-octyl-3-methyl imidazolium hexafluorophosphate IL as a binder. Nanocomposite AuNPs/GR-NH_2_ has good biocompatibility, good electron transfer capacity, a large specific surface area, and good adsorption properties [45].

### 4.3. ILs Promote the Separation of CO_2_ from GO Composite Membrane Materials

**Figure 5:**
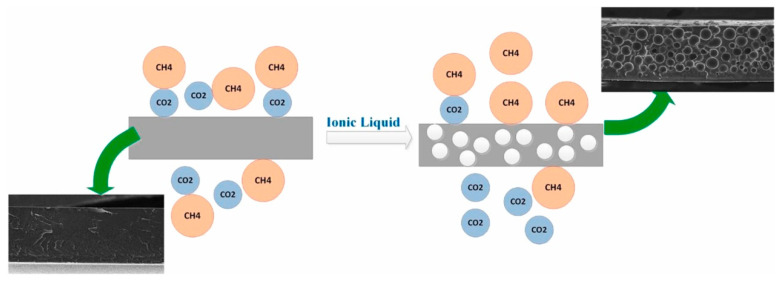
Exhibition of CO_2_ separation from CO_2_/CH_4_ mixture by using polysulfone/1-ethyl-3-methylimidazolium tetrafluoroborate composite membrane [6].

This section focuses on the properties of IL-modified membranes and their potential applications in gas separation performance. In 2017, Zhu et al. [46] supported imidazole- and pyridine-based ILs on GO catalytic materials by controlling the temperatures in aqueous solution and applied them to the cycloaddition reaction of carbon dioxide. Due to the plasticization of ILs, the addition of ILs will lead to higher porosity of the membrane [6] (Figure 5).

**Figure 6:**
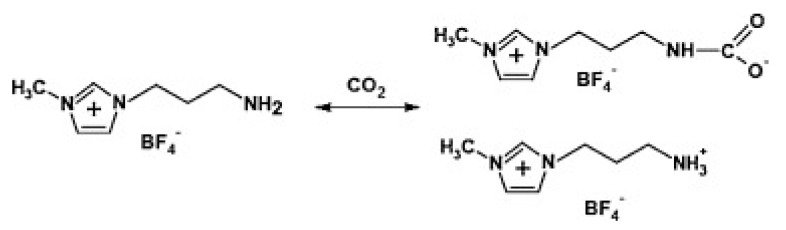
Mechanism of CO_2_ capture by NH_2_−RTIL.

**Figure 7:**
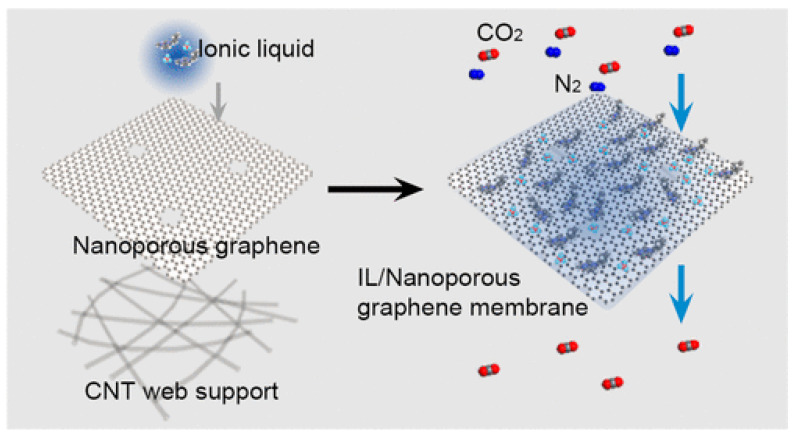
Separation of carbon dioxide and nitrogen by monolayer nanoporous graphene membrane [49].

Generally, ILs are almost non-volatile and have large CO_2_ solubilities. ILs enhance gas separation performance by contributing to the solubility selectivity of the penetrant gases. ILs form composite films in the two-dimensional nano-size channels of graphene, which changes the gas transport mechanism from Knudsen diffusion in the case of pure graphene films to solution diffusion. ILs play an important role in the separation process of CO_2_, improving the separation performance of the films [47] (Figure 6). The combination of an IL and a graphene hybrid membrane is expected to maintain the balance between permeability and selectivity in the separation process. Guo Wei et al. was able to separate CO_2_ and N_2_ by adjusting the pore size and chemical affinity of ILs while maintaining the high permeability of monolayer nanoporous graphene membranes [48] (Figure 7). The IL membrane supported by GO not only overcomes the shortcomings of poor performance of ionic liquids and inability to be reused, but it also improves the defect structure of GO. The combination of the two improves the gas separation performance. At the same time, the IL membrane supported by GO shows high temperature resistance, high durability, and high pressure stability, which provides the possibility of the real application of CO_2_ separation in practical industry in the future. Considering the toxicity of ILs, it has negligible vapor pressure, which reduces their release into the atmosphere. Atmospheric pollution due to these chemicals is unlikely.

### 4.4. ILs Enhance the Ability of Graphene Based Composites to Adsorb Heavy Metals

The agglomeration of graphene in aqueous solutions is the main factor limiting its application in adsorption processes. This limitation can be removed by using ILs to modify graphene with hydrophilic functional groups. Enhanced removal of As^3+^ and As^5+^ from aqueous solution was obtained using IL-modified magnetic graphene oxide. In this study, 1-butyl-3-methylimidazolium hexafluorophosphate ([BMIM][PF_6_]) modified magnetic graphene oxide (MGO-IL) was prepared for the first time and was used to adsorb and remove arsenic (As^3+^ and As^5+^) ions from an aqueous solution [50].

GO is a two-dimensional monomolecular layer of graphite sheet containing numerous oxygen-containing functional groups such as -OH and -COOH that prompt strong π-π electrostatic interaction with Cr^6+^ existing in chromate form in the contaminated medium. The thickness of GO is usually 1 nm, but the lateral length can extend to micrometers, providing an ultra-large specific surface area for highly efficient adsorption of heavy metal ions [51]. A. Nasrollahpour et al. prepared an IL-modified graphene oxide (IL-rGO) adsorbent and studied its chromium removal performance [52,53]. However, the high degree of oxidation lowers the specific surface area of the material. The oxygen group on GO does not form a metal complex with transition metal ions, which limits the ability of the material to absorb heavy metals. Both of these problems can be addressed by doping GO’s surface with nitrogenous groups. Similarly, in another study, aromatic nitrogen functionalities were added by grafting 1-aminopropyl-3-methylimidazolium bromide onto GO, allowing it to effectively adsorb Cr^5+^ and reduce Cr^5+^ to trivalent chromium Cr^3+^ by an indirect mechanism [54]. Luo Fang et al. used IL-functionalized graphene as an adsorbent to remove Pb^2+^ and Cd^2+^ from wastewater. The results show that functionalized graphene has great application potential in metal ion adsorption and can be reused more than 5 times [55]. Whether these materials can be recycled or reused is mostly overlooked in the literature. Hence, further research should investigate into develop sufficiently stable granular composites.

### 4.5. ILs Improve the Ability of Graphene-Based Electrodes to Degrade Pollutant PPCPs

#### 4.5.1. Characteristics and Harmful Properties of PPCPs

Daughton proposed the term PPCPs for the first time in 1999, which refers to pharmaceuticals and personal care products [56]. A series of chemical substances closely related to human activities, including all kinds of drugs (such as anti-inflammatory painkillers, blood pressure-lowering drugs, hormone drugs, antibiotic drugs, etc.), personal care products (such as soap, shampoo, toothpaste, skin care products, hair dye, etc.), and some germicidal disinfectants have wide daily life applications. In water, the residual concentration of most PPCP pollutants is in the range of ng/L-μg/L [57]. However, these pollutants have large tendency of enrichment in the environment and even at low concentrations they affect the growth of aquatic organisms, thus affecting human health [58,59]. As per the available statistics, the annual output of PPCPs is ever increasing, with the value exceeding 2 × 10^7^ tons in 2016 [60]. As a consequence of their extensive and frequent use in animal husbandry and human activities, PPCPs have become quasi-persistent pollutants via infiltration of surface water and groundwater during transformation and migration, thus threatening human health due to the intake of polluted drinking water and food. Presently, there are more than 50 kinds of PPCP compounds, such as painkillers, antineoplastic drugs, antihypertensive agents, preservatives, and polycyclic aromatic hydrocarbons, which have been detected in water environments [61]. In recent years, the presence of PPCPs in the environment has attracted considerable attention due to the improvement of environmental analysis technology along with increasing awareness of people about the environment.

Most PPCPs contain acidic or alkaline functional groups, are strongly polar, and possess biological as well as optical activity. The composition of PPCPs is complex; they are difficult to degrade, they have high biological toxicity, and their concentration level is low. Photolysis, biodegradation, adsorption, and hydrolysis are the major chemical transformations responsible for the migration and transformation of PPCPs in aquatic environments. Most PPCPs, which remain unchanged, gradually accumulate in aquatic environments after being released into sewage treatment plants, or they may also be converted into metabolites whose toxicity and harmfulness are higher than their counterparts. This results in potential biological toxicity by affecting non-target organisms in aquatic ecosystems, thus threatening the ecological environment and human health and thereby placing a huge burden on the environment [62].

The available data indicates an increasing trend for the rate of detection and concentration range of PPCPs in the urban water environments of more and more countries and regions, along with identifications of new variants of PPCPs. Different countries including the United States, Britain, Canada, Germany, Switzerland, and others have found the presence of PPCPs in the effluent of sewage treatment plants, as can be seen from Table 1.

#### 4.5.2. Electrochemical Catalysis

Presently, the traditional process for the removal of PPCPs used domestically is poor, and the degradation methods are associated with various disadvantages, such as complex operation, high energy consumption, high cost, and low mineralization. Electrochemical technology has the advantages of being simple to operate, greener in nature easier to control, etc., and thus it shows a good application prospect in water treatment. Electrochemical techniques use the reaction of pollutants on an electrode for direct degradation or indirectly oxidize and degrade organic pollutants in water via the production of active intermediates (such as ·OH,·OCl, O_3_) or via highly oxidizing metal oxides on the surface of the anode or cathode [63] so as to produce small molecular compounds such as water and carbon dioxide via direct degradation of toxic and harmful pollutants. This technology overcomes the shortcomings of advanced oxidation processes such as the requirement of high-end equipment, easy deactivation of the catalyst, high treatment cost, and low catalytic efficiency. It is easy to operate and suitable for large-scale production, and the product can be obtained in one step. It is widely used in the removal of organic pollutants from wastewater.

Electrode material is the core of electrocatalysis technology, which plays a decisive role in organic degradation and electrode stability. However, the catalytic performance of electrocatalysts is directly affected by poor dispersion of catalytic metals, easy agglomeration, and large particle size on the electrode, thus limiting the wide application of electrocatalytic technology. Therefore, reducing the cost, improving the performance, and improving the current efficiency of the electrode have become the primary challenges to be solved in the research of electrochemical oxidation [64,65].

M + H2O → M (OH) + H+ + e−

M·(OH) → MO+ H + + e−

MO + R → M + RO

M·(OH) + R→M + MCO2 + nH2O + H+ + e−

MO → M + 1/2O2

M·(OH) → M + 1/2O2 + H+ + e−

An important factor affecting electrochemical technology is the performance of the catalyst. Various catalysts such as graphene, ILs, and metal complexes have been used for the removal of PPCPs. Graphene and multi-walled carbon nanotubes were combined into an electrode for the proconcentration of low-concentration diclofenac and detected diclofenac (DCF) and examined by cyclic voltammetry (CV) and differential pulse voltammetry (DPV), which provided ideas and application value for the detection of trace levels of chemicals. The combination of graphene with multi-walled carbon nanotubes expands the electroactive surface area of the electrode and enhances the electrochemical response for the detection of DCF [66].

**Figure 8:**
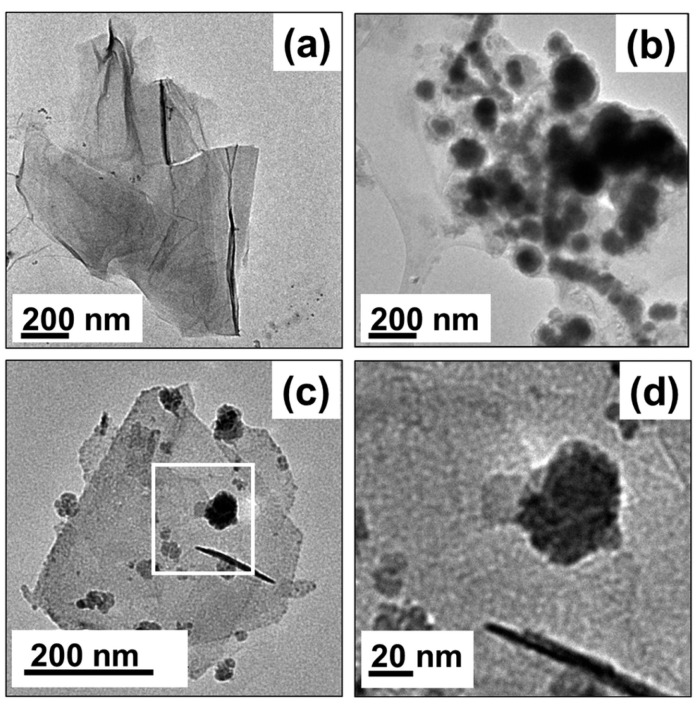
TEM images of (**a**) rGO, (**b**) nZVI, (**c**) and (**d**) rGO–nZVI NHs [67].

Graphene shows strong adsorption of PPCPs due to its highly hydrophobic surface and large theoretical specific surface area and can also be combined with other materials for further improvement of the removal efficiency of PPCPs from water. Composites of graphene and iron nanomaterials showed removal efficiency for a complex mixture of 12 kinds of PPCPs, such as antibiotic, anti-inflammatory, antiepileptic, and antidepressant drugs [67]. The physical morphology of rGO-nZVI NHs, rGO, and nZVI under TEM (Figure 8).

Wider applications of ILs as matrices are being observed for the development of amperometric sensors. They are generally used in combination with various compounds like polymers, cellulose, carbon nanotubes, metal nanoparticles, graphene, etc. Chemical reduction is used to load metal nanoparticles onto the surface of IL-functionalized graphene in order to overcome poor dispersion of the nanoparticles in the graphene matrix. The cycle life and dispersion of the polymer can be effectively improved by preventing the accumulation between the layers of reduced graphene oxide. The treatment efficiency of the electrode can be improved for the pollutants by providing more active sites for the loading of the metal catalyst [68]. Xu Dandan et al. developed a catalytic cathode consisting of CoNi/IL-rGO in the presence of imidazolium ILs, which showed oxygen reduction and hydrodehalogenation and exhibited a good removal effect for halogenated PPCPs.

A hybrid solution composed of gold nanoparticles, chitosan, 1-butyl-3-methylimidazolium tetrafluoroborate, and graphene was directly coated onto a rGO-modified glassy carbon electrode for the determination of caffeine [69]. The application of ILs for the modification of magnetic graphene nanomaterials for the removal of pollutants from real water samples is gaining increased attention. Magnetic solid-phase extraction was used to enrich and concentrate the samples. The best conditions for material synthesis and application were investigated by varying various experimental parameters, including the weighing quality of IL-modified magnetic graphene, the type and dosage of the extractant, the pH value of the aqueous solution, etc. It also has important research value and significance for sample detection and analysis. Therefore, development of new magnetic nanomaterials that have stable physical and chemical properties, larger specific surface areas, and higher precision has become the goal of research on pretreatment technology.

#### 4.5.3. Toxicity Discussion of ILs

##### Adsorption

As mentioned earlier, ILs may not directly cause atmospheric pollution. However, due to their strong solubility in water, ILs may enter the environment through wastewater. Literature reports indicated that ILs can be recovered from aqueous solution by adsorption using absorbents. Functional carbonaceous material containing carboxyl groups can be used for recovery of a water-soluble IL, such as 1-butyl-3-methyl-imidazolium chloride ([BMIM][Cl]) [70]. Activated carbon (AC) has been used as effective, environmentally friendly, and nondestructive adsorbent for the removal of various ILs in aqueous solution. Anthony et al. were the first to propose the use of AC for the adsorption recovery of [C_4_mim] [PF_6_] from wastewater [71]. The structural and chemical properties of AC can be conveniently modified for efficient adsorption via chemical treatments (acidic and basic), and modification of its physical characteristics.

##### Advanced Oxidation Processes (AOPs)

In addition to photodegradation, ultrasonic degradation, and Fenton/Fenton-like reactions, electrolytic degradation is an attractive choice [72]. In the study of Gao, J., et al., the ILs acted as both a pollutant and an electrolyte in the system, degrading imidazolium-based ILs in wastewater using plasma electrolysis [73]. Plasma electrolysis is sustained by DC or DC-pulsed glow discharges between an electrode and the electrolyte. The degradation of imidazolium-based ILs was performed by means of active radicals, such as HO radicals, H radicals, and H_2_O_2_.

## 5. Summary and Outlook

After a few years of development, the research on ILs and IL-modified graphene has become an intensively developing area (Table 2). IL/graphene composites are promising catalysts. Graphene having a high surface area and high electrical and thermal conductivity, which is conducive to the incorporation and dispersion of various active sites. In this review, IL incorporation enhances the performance of graphene in various applications, including gas separation, catalysis, and water treatment. The review article also focused on the performance of IL-graphene catalytic electrodes in practical applications. That being so, a bright future can be predicted for IL-modified electrodes.

Initially, because of its extremely low vapor pressure and low toxicity, attention was paid to how ILs could be environmentally friendly reagents. On the basis of the literature, many of them, such as BMIMPF_6_ and BMIMBF_4_, are not green. This might be ascribed to fluoride formation during the hydrolysis of [BF_4_] ^−^, which enhanced the toxicity of these substances. Considering these studies, it is sugges.ted that the properties of ILs should be related to the environment and the production of non-toxic and biodegradable ILs for the environment.

Regarding future research, IL/graphene-material composites provide a wide range of possibilities of functionalization and constitute a promising class of composites for further study. Hopefully, this review can provide some clues to support a great deal of future research on the application of ILs.

## Figures and Tables

**Table 1:** Characteristics of top 20 common PPCPs pollutants.

PPCPs	Chemical Formula	Molecular Weight	Application	Toxicity
Diclofenac	C_14_H_11_Cl_2_NO_2_	296.15	Non-steroidal anti-inflammatory drugs, anti-inflammatory, analgesic, antipyretic.	LD50 orally in mice: 390 mg/kg
Carbamazepine	C_15_H_12_N_2_O	236.27	Anticonvulsant, analgesic, anti-manic, antipsychotic.	LD50 orally in mice: 3750 mg/kg
Ibuprofen	C_13_H_18_O_2_	206.28	Non-steroidal anti-inflammatory drugs, anti-inflammatory, analgesic, antipyretic.	LD50 orally in mice: 1255 mg/kg
Triclosan	C_12_H_7_Cl_3_O_2_	289.54	Broad-spectrum antibacterial agent, used in daily chemicals.	LD50 orally in mice: 4000 mg/kg
Sulfamethoxazole	C_10_H_11_N_3_O_3_S	253.28	Broad-spectrum antibacterial agent, used in daily chemicals.	LD50 orally in mice: 3662 mg/kg
Tetracycline	C_22_H_24_N_2_O_8_	444.43	Broad-spectrum antibiotics, bacteriostatic agents.	LD50 orally in mice: 807 mg/kg
Amoxicillin	C_16_H_19_N_3_O_5_S	365.40	Broad-spectrum semi-synthetic penicillin antibiotics.	LD50 orally in mice: 25 mg/kg
Paracetamol	C_8_H_9_NO_2_	151.16	Broad-spectrum semi-synthetic penicillin antibiotics.	LD50 orally in mice: 338 mg/kg
Naproxen	C_14_H_14_O_3_	230.26	Non-steroidal anti-inflammatory drugs, anti-inflammatory, analgesic, antipyretic.	LD50 orally in mice: 1234 mg/kg
Bisphenol A	C_15_H_16_O_2_	228.29	Used as a PVC stabilizer, plastic antioxidant, UV absorber, fungicide	LC50 in rainbow trout: 3000−3500 mg/l
Ciprofloxacin	C_17_H_18_FN_3_O_3_	331.34	Quinolone antibacterial, broad antibacterial spectrum, strong and bactericidal.	LD50 orally in rats: 2000 mg/kg
Caffeine	C_8_H_10_N_4_O_2_	194.19	Astragalus alkaloid compound, central nervous system stimulant.	LD50 orally in mice: 127 mg/kg
Sulfamethazine	C_12_H_14_N_4_O_2_S	278.33	Broad spectrum bacteriostat.	LD50 i.p. in mice: 1776 mg/kg
Metoprolol	C_15_H_25_NO_3_	267.36	Treating high blood pressure and angina.	LD50 orally in mice: 2193 mg/kg
β-Estradiol	C_18_H_24_O_2_	272.38	Biochemical research, female hormone drugs	LD50 subcutaneous in rat: 300 mg/kg
Trimethoprim	C_14_H_18_N_4_O_3_	290.32	Antibacterial potentiator, broad-spectrum antibacterial	LD50 orally in mice: 7000 mg/kg
Atenolol	C_14_H_22_N_2_O_3_	266.34	Treating high blood pressure and angina.	LD50 orally in mice: 2000 mg/kg
Ofloxacin	C_18_H_2_0FN_3_O_4_	361.37	Quinolone antibacterial agent, broad-spectrum antibacterial.	LD50 orally in mice: 5290 mg/kg
Ranitidine	C_18_H_2_0FN_3_O_4_	314.40	Digestive system medication for the treatment of stomach acid, stomach ulcers.	LD50 orally in mice: 30 mg/kg
Norfloxacin	C_16_H_18_FN_3_O_3_	319.33	Quinolone antibacterial agent, broad-spectrum antibacterial.	LD50 orally in mice: 4000 mg/kg

**Table 2:** Some related literatures.

Title	Publication Date	Author
Electrochemical Method for Ease Determination of Sodium Diclofenac Trace Levels in Water Using Graphene—Multi-Walled Carbon Nanotubes Paste Electrode	2022	Sorina Motoc, Florica Manea, Anamaria Baciu, Corina Orha and Aniela Pop
Poly(ionic liquid)/graphene oxide-derived porous carbon materials as highly efcient electrocatalysts for hydrogen evolution reaction	2022	Chenming Liu, Honghong Song, Zhifeng Dai, Yubing Xiong
Composites of porous materials with ionic liquids: Synthesis, characterization, applications, and beyond	2022	Ozce Durak, Muhammad Zeeshan, Nitasha Habib, Hasan Can Gulbalkan, Ala Abdulalem Abdo Moqbel Alsuhile et al.
Functional Ionic Liquids Decorated Carbon Hybrid Nanomaterials for the Electrochemical Biosensors	2021	Pushpesh Ranjan, Shalu Yadav, Mohd Abubakar Sadique Raju Khan, Jamana Prasad Chaurasiaand Avanish Kumar Srivastava
Efficient tetracycline removal from aqueous solutions using ionic liquid modified magnetic activated carbon (IL@mAC)	2021	Edris Bazrafshan, Amin Allah Zarei, Leili Mohammadi et al.
Oxidized Graphene in Ionic Liquids for Assembling Chemically Modified Electrodes: A Structural and Electrochemical Characterization Study	2021	Valentini, F, Roscioli, D, Carbone, M, Conte, V, Floris, B, Palleschi, G, Flammini, R, Bauer, EM, Nasillo, G, Caponetti, E
Progress in the functional modification of graphene/graphene oxide: a review	2020	Wang Yu, Li Sisi, Yang Haiyan and Luo Jie
Specifying the Effects of Functionalization of Highly Reduced Graphene Oxide by an Ionic Liquid on Supercapacitive Features	2020	Mohammad Bagher Bakhshandeh and Elaheh Kowsari
Amine-Terminated Ionic Liquid Modified Magnetic Graphene Oxide (MGO-IL-NH2): A Highly Efficient and Reusable Nanocatalyst for the Synthesis of 3-Amino Alkylated Indoles	2020	Charu Garkoti, Javaid Shabir, and Subho Mozumdar
Adsorption and advanced oxidation of diverse pharmaceuticals and personal care products (PPCPs) from water using highly efficient rGO–nZVI nanohybrids	2020	Arvid Masud, Nita G. Chavez Soria, Diana S. Aga and Nirupam Aich
Recent developments in physical, biological, chemical, and hybrid treatment techniques for removing emerging contaminants from wastewater	2020	S.F. Ahmed, M. Mofijur, Samiha Nuzhat et al.
Ionic liquid-modified reduced graphene oxide electrode material with favourable electrochemical properties	2020	Chang Dong, Yijia Yu, Xiaoling Zhang, Liyan Huang, Ying Wu, Jun Li and Zhengping Liu
Structural and electronic properties of graphene and its derivatives physisorbed by ionic liquids	2020	V.S. Anithaa, R. Shankar, S. Vijayakumar
Recent advances of ionic liquids in sample preparation	2020	Juanjuan Feng, Herman Maloko Loussala, Sen Han, Xiangping Ji, Chunying Li, Min Sun
Cellulose nanocrystals/graphene oxide composite for the adsorption andremoval of levofloxacin hydrochloride antibiotic from aqueous solution	2020	Junhong Tao, Jie Yang, Chengxiao Ma, Junfeng Li et al.
Imidazole polymerized ionic liquid as a precursor for an iron-nitrogen-doped carbon electrocatalyst used in the oxygen reduction reaction	2020	Bing Han, Shuping Yu, Zhongming Wang, Hong Zhu
Ionic liquid-modified composites for the adsorptive removal of emerging water contaminants: A review	2019	Ali Ayati, Sara Ranjbari, Bahareh Tanhaei, Mika Sillanpää
Amine-terminated ionic liquid modified graphene oxide/copper nanocomposite toward efficient lubrication	2019	Chaoliang Gana, Ting Lianga, Wen Lia, Xiaoqiang Fana, Minhao Zhu
New Directions in Using Ionic Liquids in Analytical Chemistry. 2: Electrochemical Methods	2019	I. V. Pletnev, S. V. Smirnova, and N. V. Shvedene
Ionic Liquid Immobilized on Graphene-Oxide-Containing Palladium Metal Ions as an Efficient Catalyst for the Alkoxy, Amino, and Phenoxy Carbonylation Reactions	2018	Vinayak V. Gaikwad, Vitthal B. Saptal, Kei Harada, Takehiko Sasaki, Daisuke NishioHamane, and Bhalchandra M. Bhanag
Ionic liquid and nanoparticle hybrid systems: Emerging applications	2017	Zhiqi He, Paschalis Alexandridis
Graphene oxide grafted hydroxyl-functionalized ionic liquid: A highly efficient catalyst for cycloaddition of CO_2_ with epoxides	2016	Wei-Hong Zhang, Pan-Pan He, Sheng Wu, Jie Xu, Yongxin Li, Gen Zhang, Xian-Yong Wei
Sono-assisted preparation of magnetic ferroferric oxide/graphene oxide nanoparticles and application on dye removal	2015	Guodong Jiang, Qing Chang, Fufu Yang, Xiaoyun Hu, Heqing Tang
Facile fabrication of a novel anisotropic gold nanoparticle–chitosan–ionic liquid/graphene modified electrode for the determination of theophylline and caffeine	2014	Guangming Yang, Faqiong Zhao, Baizhao Zeng

## Data Availability

Not applicable.
